# Diversity Statements and Pediatric Residency Program and Department Characteristics

**DOI:** 10.1001/jamanetworkopen.2025.13013

**Published:** 2025-05-30

**Authors:** Zoie C. Sheets, Mytien Nguyen, Blake Hardin, Gabriel Abrams, Graham Gilliam, Sarah Hughes, Lisa M. Meeks

**Affiliations:** 1 Department of Pediatrics, University of Chicago, Chicago, Illinois; 2 Department of Medicine, University of Chicago, Chicago, Illinois; 3 Yale School of Medicine, New Haven, Connecticut; 4 University of Michigan Medical School, Ann Arbor; 5 Department of Medical Education, University of Illinois College of Medicine, Chicago; 6 Department of Family Medicine, University of Michigan Medical School, Ann Arbor

## Abstract

This cross-sectional study evaluates whether certain pediatric residence program characteristics are associated with the presence of a diversity statement on the program website.

## Introduction

Focus on recruiting diverse trainees has increased^1^ alongside recognition of the benefits of identity-concordant care, including improved patient satisfaction and outcomes.^2^ Diversity statements not only confirm a program’s support and inclusion of learners, but are also one mechanism for communicating a program’s commitment to diversity, equity, and inclusion (DEI) and may guide leadership decision-making. While studies have examined diversity statements across specialties such as surgery,^3^ there are no pediatric-specific studies. In this cross-sectional study, we investigate the association between pediatric residency program characteristics, including departmental factors, and diversity statements.

## Methods

Between June and October 2023, 5 team members (B.H., Z.C.S., G.A., G.G., S.H.) retrieved information from 208 pediatric residency program websites identified by the Electronic Residency Application Service. A diversity statement available on the residency program or pediatric department website; program type (as defined by the American Association of Medical Colleges); gender minority status of program director or department chair (eg, woman or nonbinary as determined using stated personal pronouns and institutional photographs); presence of a DEI faculty position at the program, department, or institution level; and National Institutes of Health (NIH) funding as a proxy for institutional prestige were evaluated. Diversity statements were assessed for inclusion of gender, race and ethnicity, sexual orientation, and disability. Discrepancies were resolved by a sixth researcher (L.M.M.). Descriptive analysis summarized the presence of a diversity statement by faculty leadership demographic, program type, and departmental NIH funding. An inclusion score reflected the number of unique marginalized identities (gender, race and ethnicity, sexual orientation, and disability; scored 2 for program or department level and 1 for institution level; score range, 0-8) included in diversity statements. Analysis of variance compared inclusion scores by program characteristics. Statistical analyses were conducted using GraphPad Prism version 9 (Dotmatics). A 2-tailed *P* < .05 was considered statistical significant. This cross-sectional study followed the STROBE guidelines and was approved by the University of Michigan institutional review board with an exemption for informed consent because it was not considered human participant research.

## Results

Among the 208 programs assessed, 102 (49.0%) were university affiliated. Overall, 117 programs (56.2%) included a diversity statement on the program or department website, and 70 programs (33.7%) included a diversity statement on the affiliated hospital’s or institution’s website (Table 1). A higher proportion of university-affiliated programs had diversity statements than those without (98.0% vs 82.1%; *P* < .001). A higher proportion of programs with DEI-specific faculty positions within the program and department or hospital had a diversity statement compared with programs without (program, 100.0%; department or hospital, 97.0%; without, 70.0%; *P* < .001). A higher proportion of NIH-funded programs had diversity statements than those without (96.9% vs 83.6%; *P* < .001).

**Table 1.** Characteristics of Diversity Statements Among Pediatric Programs and Departments

Characteristic	Programs and departments, No. (%)				*P* value
	Total (N = 208)	Diversity statement^a^			
		No (n = 21)	Program or department level (n = 117)	Institution or hospital level (n = 70)	
Program type					
University affiliated	102 (49.0)	2 (9.5)	71 (60.7)	29 (41.4)	<.001
Other	106 (51.0)	19 (90.5)	46 (39.3)	41 (58.6)	
Gender identity of department leadership^b^					
Non–gender minority	41 (19.8)	3 (14.3)	18 (15.5)	20 (28.6)	.08
Gender minority	166 (80.2)	18 (85.7)	98 (84.5)	50 (71.4)	
Presence of DEI faculty^c^					
None	60 (28.8)	18 (85.7)	20 (17.1)	22 (31.4)	<.001
Program level	47 (22.6)	0 (0.0)	43 (36.8)	4 (5.7)	
Department or hospital level	101 (48.6)	3 (14.3)	54 (46.2)	44 (62.9)	
Departmental NIH funding					
No	110 (52.9)	18 (85.7)	51 (43.6)	41 (58.6)	<.001
Yes	98 (47.1)	3 (14.3)	66 (56.4)	29 (41.4)	

Abbreviations: DEI, diversity, equity, and inclusion; NIH, National Institutes of Health.

^a^ Statements of nondiscrimination were not accepted as proxies for diversity statements.

^b^ Whether the program director and/or department chair is a gender minority. Presumed gender identity was determined by the combined use of personal pronouns featured on program websites and institutional photographs.

^c^ A faculty member whose role is dedicated to promoting DEI, eg, associate director or director of DEI.

Among programs with diversity statements, gender, race and ethnicity, sexual orientation, and disability were included in 47 (22.6%), 70 (33.6%), 45 (21.6%), and 30 (14.4%) programs at the program or department level, respectively, and 31 (14.9%), 38 (18.3%), 29 (13.9%), and 26 (12.5%) at the institution level. University-affiliated programs had higher inclusion scores than those not affiliated (mean [SD], 3.0 [3.1] vs 2.0 [2.6]; *P* = .01) (Table 2). Programs with a DEI faculty at the program or institution level and those with NIH funding had significantly higher inclusion mean (SD) scores than those without (DEI faculty at program level, 3.6 [3.4]; at institution level, 2.7 [2.8]; without DEI faculty, 1.1 [2.2]; *P* < .001; with NIH funding, 2.9 [3.1]; without, 2.0 [2.7]; *P* = .03). There was no significant association between gender minority leadership and inclusion scores (Table 2).

**Table 2.** Inclusion Score Within DEI Statements, by Pediatric Program and Department Characteristics

Characteristic	Inclusion score, mean (SD)	*P* value
Overall	2.5 (2.9)	NA
Program type		
University affiliated	3.0 (3.1)	.01
Other	2.0 (2.6)	
Gender identity of department leadership		
Non–gender minority	2.0 (2.5)	.24
Gender minority	2.6 (3.0)	
Presence of DEI faculty^a^		
None	1.1 (2.2)	<.001
Program level	3.6 (3.4)	
Department or hospital level	2.7 (2.8)	
Departmental NIH funding		
No	2.0 (2.7)	.03
Yes	2.9 (3.1)	

Abbreviations: DEI, diversity, equity, and inclusion; NA, not applicable; NIH, National Institutes of Health.

^a^ A faculty member whose role is dedicated to promoting DEI, eg, associate director or director of DEI.

## Discussion

In this study, 56% of pediatric residency programs included a diversity statement at the program or department level, most often referencing race and ethnicity. University-affiliated and NIH-funded programs as well as those with DEI-specific faculty roles had higher overall inclusion scores. While diversity statements alone may not serve as a proxy for inclusion, prior studies have shown that applicants value program diversity support in ranking decisions, especially women and members of racial minority groups.^4^

Our findings suggest that the presence of a DEI-specific role in departmental leadership is associated with inclusivity in diversity statements. Diverse leadership improves patient outcomes,^5^ making workforce diversity essential. Despite these associations, women and faculty who belong to minoritized racial and ethnic groups remain underrepresented in tenured faculty positions in pediatric departments.^6^

This study’s limits include the use of publicly available websites, which may not reflect internal practices or evolving commitments and limit our capacity to fully understand program leaderships’ identities. DEI-specific faculty roles and visible commitments to diversity are essential steps toward building a supportive and equitable training environment that benefits all trainees and promotes a diverse pediatric workforce.

## Notes

Supplement.
Data Sharing Statement
