# Supplementary material for: Diversity Statements and Pediatric Residency Program and Department Characteristics
Source: JAMA Netw Open. 2025 May 30;8(5):e2513013. doi: 10.1001/jamanetworkopen.2025.13013 (PMC12125634; doi:10.1001/jamanetworkopen.2025.13013)
Supplement: Supplement. — Data Sharing Statement [file jamanetwopen-e2513013-s001.pdf]

Data Sharing Statement

Sheets. Diversity Statements and Pediatric Residency Program and Department Characteristics. *JAMA Netw Open*. Published May 30, 2025. doi:10.1001/jamanetworkopen.2025.13013

Data
Data available: No

Additional Information
Explanation for why data not available: Our IRB does not allow for data sharing
